# Biosynthetic novelty index reveals the metabolic potential of rare actinobacteria isolated from highly oligotrophic sediments

**DOI:** 10.1099/mgen.0.000921

**Published:** 2023-01-20

**Authors:** Luz A. González-Salazar, Michelle Quezada, Lorena Rodríguez-Orduña, Hilda Ramos-Aboites, Robert J. Capon, Valeria Souza-Saldívar, Francisco Barona-Gomez, Cuauhtémoc Licona-Cassani

**Affiliations:** ^1^​ Industrial Genomics Laboratory, Escuela de Ingeniería y Ciencias, Tecnológico de Monterrey, Nuevo León, Mexico; ^2^​ Institute for Molecular Bioscience, University of Queensland, St Lucia, Queensland 4072, Australia; ^3^​ Evolution of Metabolic Diversity Laboratory, Unidad de Genómica Avanza (LANGEBIO), Cinvestav-IPN, Irapuato, Mexico; ^4^​ Departamento de Ecología Evolutiva, Instituto de Ecología, Universidad Nacional Autónoma de México, Ciudad de México, Mexico; ^5^​ Division of Integrative Biology, Institute for Obesity Research, Tecnológico de Monterrey, Nuevo León, Mexico; ^†^​ Present address: Microbial Diversity and Specialized Metabolism Laboratory, Institute of Biology, Leiden University, Leiden, Netherlands

**Keywords:** *Actinokineospora*, BiNI, Cuatro Cienegas, genome mining, *Lentzea*, natural products

## Abstract

Calculations predict that testing of 5 000–10 000 molecules and >1 billion US dollars (£0.8 billion, £1=$1.2) are required for one single drug to come to the market. A solution to this problem is to establish more efficient protocols that reduce the high rate of re-isolation and continuous rediscovery of natural products during early stages of the drug development process. The study of ‘rare actinobacteria’ has emerged as a possible approach for increasing the discovery rate of drug leads from natural sources. Here, we define a simple genomic metric, defined as biosynthetic novelty index (BiNI), that can be used to rapidly rank strains according to the novelty of the subset of encoding biosynthetic clusters. By comparing a subset of high-quality genomes from strains of different taxonomic and ecological backgrounds, we used the BiNI score to support the notion that rare actinobacteria encode more biosynthetic gene cluster (BGC) novelty. In addition, we present the isolation and genomic characterization, focused on specialized metabolites and phenotypic screening, of two isolates belonging to genera *Lentzea* and *Actinokineospora* from a highly oligotrophic environment. Our results show that both strains harbour a unique subset of BGCs compared to other members of the genera *Lentzea* and *Actinokineospora *. These BGCs are responsible for potent antimicrobial and cytotoxic bioactivity. The experimental data and analysis presented in this study contribute to the knowledge of genome mining analysis in rare actinobacteria and, most importantly, can serve to direct sampling efforts to accelerate early stages of the drug discovery pipeline.

## Data Summary

1. Genome sequences analysed in this study (Table 1) have been previously deposited in the National Center for Biotechnology Information (NCBI) with the accession numbers listed in Table S1 (available with the online version of this article).
2. *
Lentzea* sp. CC55 and *Actinokineospora* sp. PR83 genome sequences produced in this study have been deposited at GenBank/ENA/DDBJ under the accession numbers JAJCXE000000000 and JAJCXD000000000, respectively.
3. Environmental *Streptomyces* 8S0 sequences analysed in this study have been deposited at GenBank/ENA/DDBJ under the accession numbers CP045031, JAJPUG000000000, JAJPUH000000000 and JAJPUI000000000.

**Table 1.** Isolation and genomic information of the *Actinokineospora* and *Lentzea* strains used in this study

Genome name	Source	No. of contigs	N_50_	Length (Mb)	G+C (mol %)	Reference
***Lentzea*** **genomes**						
* L. waywayandensis* strain DSM 44232	Soil, Waywayanda Lake, USA	39	475 994	10.15	68.9	[83]
* L. alba* strain NEAU.D13	Soil, Aohan Banner, Chifeng, Mongolia	42	431 430	10.21	68.7	[84]
* L. albidocapillata* subsp. *violacea* strain DSM 44796	Soil, gold mine cave in Kongju, Korea	58	331 077	8.67	69	[85]
* L. flaviverrucosa* strain CGMCC 4.578	Soil, Shanxi Province, China	34	409 948	9.47	69.2	[86]
* L. jiangxiensis* strain CGMCC 4.6609	Soil, Jiangxi Province, South-East China	62	279 756	8.59	70.2	[53]
* L. albida* strain DSM 44437	Soil, Jiangxi Province, South-East China	43	352 714	9.44	70.2	[83]
* L. albidocapillata* strain DSM 44073	Tissue specimen, Germany	43	397 314	8.64	68.7	[87]
* L. flaviverrucosa* strain DSM 44664	Soil, Shanxi Province, China	38	462 737	9.47	69.2	[86]
* L. terrae* strain NEAU-LZS 42	Soil, Henan Province, China	145	211 542	10.58	68.6	[88]
* L. albidocapillata* strain NRRL B-24057	Tissue specimen, Germany	127	330 024	8.64	68.7	[87]
* L. pudingi* strain CGMCC 4.7319	Limestone, South-West China	63	432 100	9.21	69.1	[89]
* L. kentuckyensis* strain NRRL B-24416	Equine placenta, USA	317	92 128	10.21	68.8	[90]
* Lentzea* sp. strain FXJ1.1311	Soil, Tibet, China	120	199 708	9.37	69.5	[68]
* L. guizhouensis* strain DHS C013	Limestone, Guizhou Province, South-West China	1	9 997 872	9.99	70	[69]
* L. cavernae* strain CGMCC 4.7367	Limestone, Guizhou Province, South-West China	43	472 751	9.74	69.6	[26]
* L. aerocolonigenes* NBRC 13195	Soil, Nagasaki City	61	348 916	10.69	69	not found
* L. atacamensis* DSM 45479	Soil, Atacama Desert, Northern Chile	38	785 641	9.31	68.9	[91]
* L. nigeriaca* DSM 45680	Soil, Abuja, Nigeria	42	641 913	9.33	68.4	[56]
* L. fradiae* CGMCC 4.3506	Soil, Wutaishan Mountain, Shanxi Province, China	47	479 571	8.51	70.5	[92]
* L. deserti* DSM 45480	Soil, Salar de Atacama, Atacama Desert, Chile	50	442 485	9.53	68.8	[91]
* L. xinjiangensis* CGMCC 4.3525	Soil, Xinjiang, North-West China	61	283 838	8.69	68.9	[93]
* L. flava* JCM 3296	Soil, Acharya Nagarjuna, India	190	133 734	9.72	69	[94]
* L. aerocolonigenes* NRRL B-3298	Soil, Nagasaki City, Japan	204	163 428	10.65	69	[95]
* Lentzea* sp. CC55 *	Sediment, Cuatro Cienegas, Coahuila, México	101	145 251	8.27	70.5	This study
***Actinokineospora*** **genomes**						
* A. enzanensis* strain DSM 44649	Forest soil, Yamanashi, Japan	95	161 170	8.12	70.8	[65]
*A. xionganensis* HBU206404	Xiongan lakeside soil, China	70	325 226	6.81	69.6	[58]
* A. terrae* strain DSM 44260	Soil, Yamanashi Prefecture, Japan	41	383 933	7.59	71	[66]
* A. bangkokensis* strain DSM 44EHW	Rhizospheric soil, Bangkok, Thailand	78	168 142	7.45	74	[96]
* A. mzabensis* strain CECT 8578	Soil, Saharan desert, South Algeria	32	442 119	7.55	72.8	[60]
* A. auranticolor* strain YU 961–1	Soil, Yamanashi and Nagano Prefectures, Japan	61	339 969	8.43	71.8	[65]
* A. inagensis* strain DSM 44258	Fallen leaves, Lake Inaga, Yamanashi, Japan	120	109 727	7.28	70.2	Not found
* A. spheciospongiae* strain EG49	Marine sponge, Germany	263	52 481	7.53	72.8	[54]
* A. cianjurensis* strain DSM 45657	Leaf-litter, Indonesia	19	1 225 445	7.62	70.9	[97]
* A. pegani* strain TRM65233	Root from *Peganum harmala*, China	137	104 380	6.44	72.6	[59]
* A. fastidiosa* strain JCM 3276	Soil, Egypt	30	1 778 554	7.04	72.5	[98]
* A. alba* DSM 45114	Soil, Xinjiang Province, China	1	7 288 615	7.29	69.7	[62]
* A. baliensis* DSM 45656	Soil and plant-litter, Indonesia	1	7 681 387	7.68	70.6	[97]
* A. iranica* IBRC-M 10403	Soil, Inche-Broun hypersaline wetland, North Iran	44	291 113	6.58	70.8	[70]
* A. alba* CPCC 201030	Soil, Xinjiang Province, China	35	459 069	7.27	69.7	[62]
* A. alba* IBRC-M 10655	Soil, Xinjiang Province, China	34	443 362	7.27	69.7	[62]
* Actinokineospora* sp. PR83*	Sediment, Cuatro Cienegas, Coahuila, México	182	77 654	7.64	72.7	This study

**Impact Statement**

We define a novel genomic metric, defined as biosynthetic novelty index (BiNI), that can be used to rapidly rank strains according to the novelty of the subset of encoding biosynthetic clusters. We used the BiNI score to prioritize a subset of potential natural product producers from different taxonomic groups and ecological contexts. We demonstrate the applicability of the BiNI score using two rare actinobacteria strains isolated from a highly oligotrophic environment. Our genomic analysis includes a deep biosynthetic gene cluster comparison for the rare actinobacteria *Lentzea* and *Actinokineospora *.

## Introduction

A period of 10 to 12 years has been estimated for a natural product (NP) to become a drug available in the market. Expenses during this period of multi-stage and interdisciplinary research can exceed US$1 billion (£0.8 billion, £1=$1.2) per molecule [1]. The first step of the drug discovery process begins when entire collections of thousands of microbial isolates are subjected to different fermentation conditions to generate libraries of NPs that need to be tested against the drug target. Overall, from 5 000 to 10 000 NPs that show promising *in vitro* activity against a given target, 5 will enter clinical testing and only 1 will be registered for marketing and commercialization [2]. Increasing the discovery rate of microbial isolates that generate positive results against drug targets would directly impact the overall efficiency and costs of the entire drug discovery process [3, 4].

Microbes are one of the most prolific sources of bioactive NPs. Specifically, members of the phylum '*Actinobacteria'* produce approximately 10 000 different bioactive molecules, which account for approximately 45 % of all microbial metabolites described to date [5]. The wide chemical diversity found in actinobacteria includes polyketides (PKs) [6], non-ribosomal peptides (NRPs) [7], hybrid PK/NRPs [8], terpenes [9], ribosomally synthesized and post-translationally modified peptides (RiPPs), among other representative chemical families [10]. While chemically diverse, NPs are produced within discrete and highly conserved genomic regions (biosynthetic gene clusters, BGCs). Recently, genome sequencing and bioinformatic tools have allowed genomic screening and predictions of the gene-structure associations of NPs [11]. However, traditional bioactive-guided approaches using microbes and plants remain hampered by low production and high rediscovery rates [12]. These limitations can be addressed by searching for less characterized microbial species in highly oligotrophic ecosystems (i.e. environments with low concentrations of nutrients, such as deep oceanic sediments, caves, glacial and polar ice, and deep subsurface soil among others) [13, 14].

The term rare actinobacteria was first defined as slow-growing actinobacteria found with lower frequency, compared to the frequency of isolation of *Streptomyces* using conventional methods [13, 15, 16]. Since then, rare actinobacteria (or underisolated actinobacteria) are known as a genetic repository of novel BGCs [14]. For example, *Pseudonocardia* sp. HS7, isolated from the cloacal aperture of sea cucumber (*Holothuria moebii*), can produce one of the curvularin macrolides that have shown potential cytotoxicity toward six tested cancer cell lines [17]. The endophytic actinomycete *Micromonospora* sp. GMKU326 isolated from the root of a leguminous plant, Maklam phueak (*Abrus pulchellus* Wall. Ex Thwaites subsp. *pulchellus*), produces maklamicin, a new PK with promising antimicrobial activity against Gram-positive pathogens [18]. Moreover, isolates from soil samples taken in a rainforest undergoing restoration contained new polycyclic antibiotics such as pradimicin-IRD, produced by *Amycolatopsis* sp. IRD-009, which presented antimicrobial activity against *Streptococcus agalactiae *, *Pseudomonas aeruginosa* and *Staphylococcus aureus* [19]. As a result of taxonomically selective isolation and genetic techniques, the number of rare genera increased to 220, with 2 500 bioactive metabolites, according to reports up to 2010 [13, 20–22].

In recent years, highly oligotrophic environments [23] have generated interest as a rich source of rare actinobacteria [24–28]. The Cuatro Cienegas Basin, located in the Chihuahuan Desert in the State of Coahuila, Mexico, has been mostly studied due to the low nutrient availability in the soil [29]. This ecosystem shows a nutrient imbalance in nitrogen-to-phosphorus stoichiometry (N:P 157 : 1), with a high calcium accumulation rising from 35 to 90 % across the microbialite matrix [30, 31]. Nutrient limitation, for example, the extremely low concentrations of phosphate and iron, generates the environmental conditions to modulate unique metabolic traits in diverse microbial communities [32–34]. Evidence of the potential for novel chemical diversity in the Cuatro Cienegas Basin was reported in two strains of *Micrococcus* sp. (strain CH3 and CH7) for which phylogenomic analysis and siderophore production profiles demonstrate the production of previously unreported aryl-substituted desferrioxamines [33].

Here, we present the implementation of a simple genomic metric (biosynthetic novelty index, BiNI) that allows direct classification of microbial strains according to their biosynthetic originality. We use the BiNI score to perform a strain prioritization exercise of members of different taxonomic families (and different ecological contexts), including those of rare actinobacteria. We demonstrate the potential application of the BiNI score by isolating and comparing the biosynthetic potential of two rare actinobacteria belonging to the genera *Lentzea* and *Actinokineospora* of the Cuatro Cienegas Basin. Finally, using phylogenomics and different genome mining bioinformatic tools, we report the scope of the biosynthetic potential of our isolates and all of the members from these two genera reported to date. Overall, our results highlight the importance of exploring natural extreme environments for the research of NPs and describe an efficient method for strain prioritization according to biosynthetic novelty.

## Methods

### Strain isolation and culture conditions

*Lentzea* sp. CC55 and *Actinokineospora* sp. PR83 were isolated from the Cuatro Cienegas Basin, as previously reported [32]. *Lentzea* sp. CC55 was isolated from the region of Pozas Azules (26°49'39.01" N, 102°01'26" W), while *Actinokineospora* sp. PR83 was isolated from Pozas Rojas (26°52'16.8" N, 102°1'11.3" W). Homogenized sediments were cultured in serial dilutions on MS solid media (per litre: mannitol, 20 g; soy flour, 20 g; agar, 20 g; in tap water) [35]. All cultures were supplemented with cycloheximide (100 µg ml^−1^) and nalidixic acid (30 µg ml^−1^). Bioactivity assays were performed for both strains using the OSMAC () approach. *Lentzea* sp. CC55 and *Actinokineospora* sp. PR83 isolates were cultured in the following media (solid and liquid media): International *Streptomyces* Project (ISP)-2 [36], ISP-4 [37], R5 [38], Czapek [1 l: 1 g K_2_HPO_4_, 0.01 g FeH_14_O_11_S, 0.5 g H_14_MgO_11_S, 0.5 g KCl, 3 g NaNO_2_, 30 g sucrose, 15 g agar], BG [1 l: 0.2 g MgSO_4_, 0.2 g CaCl_2_, 1 g KH_2_PO_4_, 1 g K_2_HPO_4_, 1 g (NH_4_)_2_SO_4_, 0.05 g FeCl_3_], BS [1 l: 0.2 g MgSO_4_, 0.02 g CaCl_2_, 1 g KH_2_PO_4_, 1 g K_2_HPO_4_, 1 g (NH_4_)_2_SO_4_, 0.05 g FeCl_3_], M1 [1 l: 10 g soluble starch, 4 g yeast extract, 2 g peptone, 20 g NaCl, 18 g agar] and oatmeal [1 l: 20 g oatmeal, 0.001 g Fe_2_H_14_O_19_S_3_, trace salts, 0.001 g Cl_2_H_8_MnO_4_, 0.001 g ZnSO_4_. 7H2O, 18 g agar]. The ISP-2, BG, BS, R5 and M1 media were prepared using glucose purchased from Biochemicals (Australia); yeast extract from Merck; peptone from Oxoid; and soluble starch, malt extract, mannitol, sucrose and agar from Sigma-Aldrich. ISP-4 and oatmeal media were purchased from DB Difco (Fisher-Scientific).

### Genome sequencing and assembly

Genomic DNA was extracted from 15 ml ISP-2 liquid cultures (spore concentration of 1×10^6^ spores ml^−1^). Shake flasks were inoculated with 50 µl of the spore suspension and incubated for 48 h at 28 °C, at 200 r.p.m. The biomass was transferred into a sterile porcelain dish for liquid nitrogen lysis. The crushed mycelium was mixed with 400 µl TE buffer supplemented with lysozyme (20 mg ml^−1^) (Sigma-Aldrich), 10 % (w/v) SDS and proteinase K (20 mg ml^−1^) (Fisher-Scientific), for chemical rupture. The lysate was cooled and extracted twice with equal volumes of phenol:chloroform:isoamyl alcohol (25 : 24 : 1, v/v) and phenol:chloroform (25 : 24, v/v) (Fisher-Scientific) at 10 000 r.p.m for 5 min. The aqueous phase was transferred for DNA precipitation with 70 % ethanol at −20 °C for 30 min. The pellet was formed by centrifuging at 10 000 r.p.m for 15 min. Genomic DNA was resuspended in deionized sterile water and checked for quality using 260/280 and 230/260 ratios provided by a NanoDrop 1 000 spectrophotometer (NanoDrop Technologies) and a 1 % agarose gel for electrophoresis. Whole-genome sequencing was performed using the Illumina MiSeq platform with 2×300 bp paired-end reads. The reads obtained were trimmed using Trimmomatic v0.32 [39] and assembled using Velvetg and Velveth v1.2.10 [40]. *k*-mers ranging from 31 to 171 (increasing 10 units per iteration) were tested, and the best assembly was selected for annotation and analysis.

A database of genomes from *Lentzea* (23 genomes) and *Actinokineospora* (16 genomes) was generated using publicly available genomes at the National Center for Biotechnology Information (NCBI) up to September 2021 (Table S1). The inclusion criteria were focused on the quality of the assembly. The mean number of contigs in each group was 81.2 and 66.3, for *Lentzea* and *Actinokineospora *, respectively. All genomes were annotated using the Rapid Annotations of the Subsystems Technology (rast) platform [41]. A core-/pan-genome analysis was performed for both datasets using the Bacterial Pan-Genome Analysis (bpga) tool [42] in the usearch algorithm, with the similarity threshold set to 0.5 orthologue identification. A core phylogenetic tree was reconstructed with the protein sequences of core genes from bpga. The process included split sequences for protein family, alignment with muscle v.3.8.31 [43] and trimming with the Gblocks package. The processed alignments were concatenated into a supermatrix with FasConCat [44] and finally the iq-tree software [45] was used for phylogeny reconstruction. The phylogenetic tree was visualized using FigTree v.1.4.4 (http://tree.bio.ed.ac.uk/software/figtree/). Prediction of BGCs was performed for all the genomes in both datasets using the Antibiotic and Secondary Metabolite Analysis Shell (antiSMASH), version 6.0 [46], which identifies BGCs using a signature profile hidden Markov model based on multiple sequence alignments of experimentally characterized signature proteins or protein domains. The BGC similarity networks were constructed only with complete BGCs for all *Lentzea* and *Actinokineospora *, as well as the MIBiG (Minimum Information about a Biosynthetic Gene cluster) database [47] (1802 BGCs), using the Python-based platform Biosynthetic Gene Similarity Clustering and Prospecting Engine (BiG-SCAPE) [48] with default settings. antiSMASH results were uploaded in the online repository BiG-FAM (Biosynthetic Gene Cluster Families Database) in order to search for ‘homologous’ groups of BGCs putatively encoding the production of similar specialized metabolites, through distance measurement [49].

For determination of the novelty of the biosynthetic potential encoded by a given isolate, we used a metric that mainly relates the number of clusters (*n*) identified by antiSMASH and the distance (*d*) sampling test result provided by the BiG-FAM platform (threshold value >900) with the equation BiNI=∑d/n . The BiNI represents an indicator of the novelty of the subset of BGCs encoded by each isolate and could be valuable in terms of obtaining insights into the contextual ecology or the biosynthetic potential of a given taxonomic gender.

### Chromatography-based purification and bioactivity assays

Cultures were extracted with ethylacetate:methanol (3 : 1) (30 ml) for solid media and with ethylacetate (1.5 ml) for liquid media. The organic extracts were dried and resuspended in methanol (500 μl and 50 μl for solid and liquid, respectively). The resuspended extracts were then filtered through a 0.45 µm polytetrafluoroethylene (PTFE) membrane from Sigma-Aldrich before running in the UHPLC-DAD (ultra-high-performance liquid chromatography-diode array).

Antimicrobial activities were measured against the Gram-positive bacteria *Staphylococcus aureus* (ATCC 25923) and *Bacillus subtilis* (ATCC 6633), and the Gram-negative bacteria *Escherichia coli* (ATCC 25922) and *P. aeruginosa* (ATCC 27853), using the broth micro-dilution method [50]. The test was performed (in triplicate) in 96-well microtitre plates by serial dilution in tryptic soy broth (TSB). Crude microbial extracts (1 mg ml^−1^) were tested first, then an aliquot (20 µl) was transferred followed by addition of freshly prepared microbial broth (180 µl, 104–105 c.f.u. ml^−1^ cell density) for the dilution series. The plates were incubated at 26.5 °C for 24 h. The optical density of each well was measured spectrophotometrically at 600 nm using a POLARstar Omega plate reader (BMG LABTECH). Both media with and without microbial inoculation were tested as negative controls.

Cytotoxicity assays were carried out using the MTT [3-(4,5-dimethylthiazol-2- yl)−2,5-diphenyltetrazolium bromide; Sigma-Aldrich] method, as previously described [51], using adherent NCIH460 (human large cell lung carcinoma). Cells were harvested with trypsin (from Sigma-Aldrich) and dispensed into 96-well microtitre assay plates at 2 000 cells per well and incubated for 18 h at 37 °C with 5 % CO_2_ (to allow cells to attach). Crude extracts were dissolved in 5 % DMSO in PBS (v/v) and aliquots (20 µl) tested at a final concentration of 1 mg ml^−1^. Vinblastine was used as positive control and negative control wells were treated with 5 % aqueous DMSO. After 68 h incubation at 37 °C with 5 % CO_2_, an aliquot (20 µl) of MTT in PBS (4 mg ml^−1^) was added to each well (final concentration of 0.4 mg ml^−1^), and the microtitre plates incubated for a further 4 h at 37 °C with 5 % CO_2_. Following this final incubation, the medium was aspirated and the formazan crystals that precipitated were dissolved in DMSO (100 µl per well). The absorbance for each well was measured at 580 nm on a POLARstar Omega microtitre plate reader. IC_50_ values were calculated using Prism 5.0 (GraphPad Software), as the concentration of analyte required to achieve 50 % inhibition of cancer cell growth (compared to negative controls). All experiments were performed in duplicate.

## Results

### BiNI

**Fig. 1.**
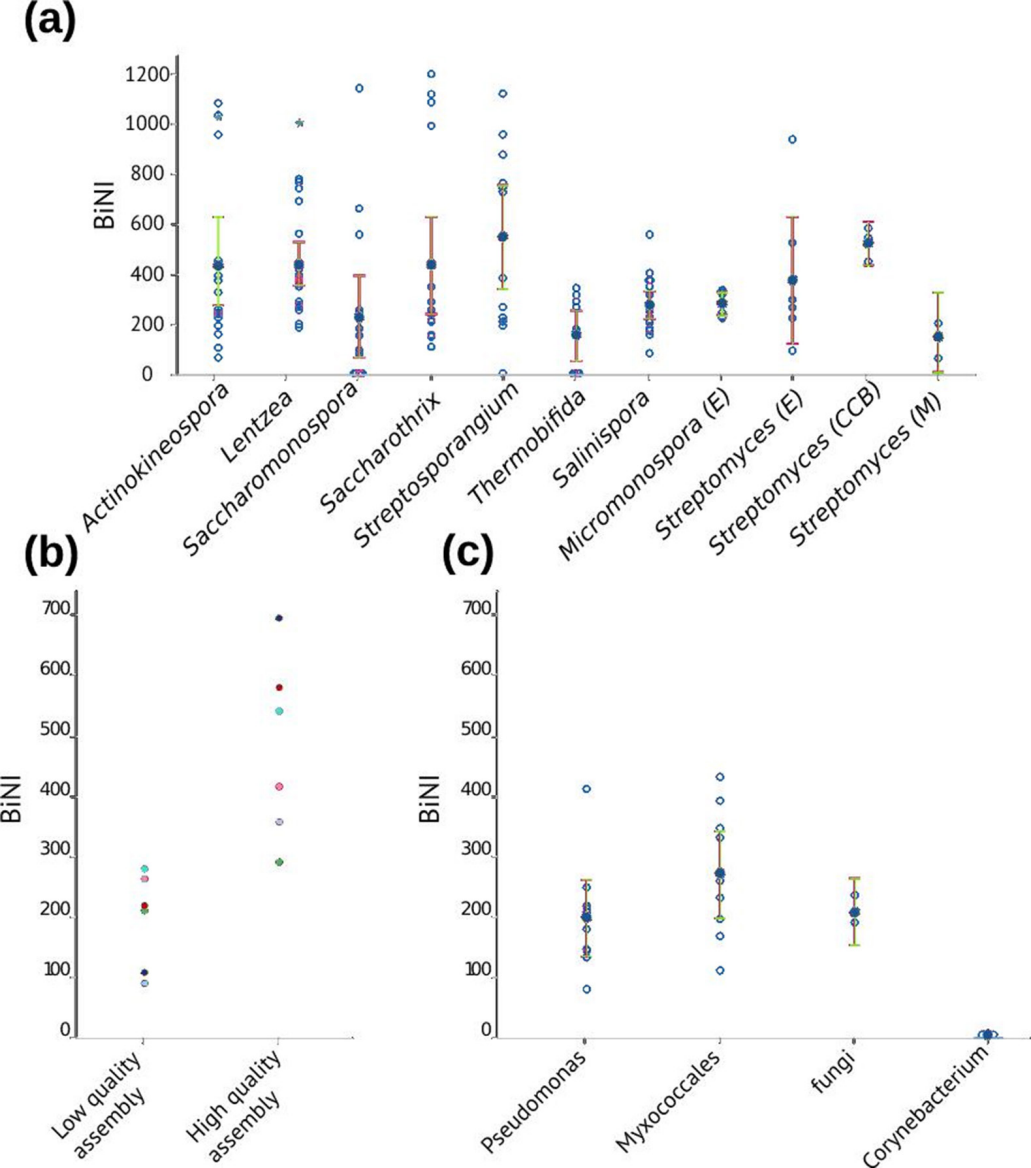
BiNI of different actinobacteria genera. (a) BiNI comparison between genomes from rare genera of actinobacteria (* Actinokineospora *, *Lentzea *, *Saccharomonospora *, *Saccharothrix *, *Streptosporangium *, *Thermobifida and Salinispora)*; endophytic actinobacteria (E) (* Micromonospora* and *Streptomyces *); free-living *Streptomyces* isolated from Cuatro Cienegas Basin (CCB); and reference *Streptomyces (M)* (*Streptomyces xiamenensis 318*, *Streptomyces rapamycinicus* NRRL 549 and *Streptomyces coelicolor* A32). Mean values (solid blue) are reported for all groups. Blue stars represent the BiNI values of *Actinokineospora* sp. PR83 and *Lentzea* sp. CC55 isolated in this study from the Cuatro Cienegas Basin. (b) BiNI comparison between low-quality and high-quality genome assemblies. Blue, *Streptomyces* sp. KL122B (2104 vs 18 contigs); red, *Streptomyces* sp. CC228A (1299 vs 26 contigs); green, *Streptomyces* 8s0 CC219A (1172 vs 382 contigs); purple, *Streptomyces* sp. KL111A (2096 vs 205 contigs); cyan, *Streptomyces* 8s0 CC210A (847 vs 9 contigs); pink, *Streptomyces* sp. CC201C (742 vs 23 contigs). (c) A comparison of the BiNI of reference strains, including genomes from *Pseudomonas *, *Myxococcales *, fungi and *Corynebacterium *.

We first used a database of high-quality genomes including common and rare actinobacteria (underisolated actinobacteria) from public databases and our strain collection (Table S1). We estimated the BiNI for all the genomes and compared the taxonomic genera recognized as rare actinobacteria, including *Actinokineospora* (17 genomes), *Lentzea* (24 genomes), *Saccharomonospora* (14 genomes), *Saccharothrix* (17 genomes), *Streptosporangium* (14 genomes), *Thermobifida* (10 genomes) and *Salinispora pacifica* strains (18 genomes) (Fig. 1a). Taking into account the entire genera, *Streptosporangium* showed the highest novelty index (BiNI=548.4), followed by *Actinokineospora* (BiNI=451.6), *Lentzea* (BiNI=439.2) and *Saccharothrix* (BiNI=438.4). However, *Saccharomonospora *, *Thermobifida* and *Salinispora* showed the lowest novelty BiNIs (155.2, 231.1 and 274, respectively). While detailed information about the isolation site is missing from databases, we observed that most of highest BiNI-scored rare actinobacteria were isolated from desert soils. For example, *Streptosporangium saharense* strain CECT 8840 (Saharan soil, BiNI=1120), *Actinokineospora fastidiosa* JCM 3276 (Egyptian soil, BiNI=1 097), *Actinokineospora* sp. PR83 (Cuatro Cienegas, BiNI=1049), *Lentzea* sp. CC55 (Cuatro Cienegas, BiNI=1 008) and *Saccharothrix tamanrassetensis* CECT8640 (Saharan soil, BiNI=1122). We did not observe a specific environment of isolation for strains with low BiNI.

In addition, we tested the robustness of the BiNI by assessing genomes of different assembly quality and length (Fig. 1b). We compared the BiNIs of highly fragmented genome assemblies of five *Streptomyces* isolates (sequenced with the Illumina platform) with their corresponding high-quality assemblies obtained by Oxford Nanopore sequencing: *Streptomyces* sp. KL122B (2104 vs 18 contigs), *Streptomyces* sp. CC228A (1299 vs 26 contigs), *Streptomyces* 8s0 CC219A (1172 vs 382 contigs), *Streptomyces* sp. KL111A (2096 vs 205 contigs), *Streptomyces* 8s0 CC210A (847 vs 9 contigs) and *Streptomyces* sp. CC201C (742 vs 23 contigs). We observed that the biosynthetic novelty is underestimated when it is tested with low-quality genome assemblies. Finally, we tested the BiNI of genomes from well-characterized strains, which are not expected to show a high BiNI, including *Pseudomonas *, *Corynebacterium *, *Myxococcales* and fungi (Fig. 1c).

### Oligotrophic environments harbour strains with high BiNI

We isolated *Lentzea* sp. CC55 and *Actinokineospora* sp. PR83 from sediments of the Cuatro Cienegas Basin, Mexico. Growth on MS agar showed circular colonies with raised elevation and filiform margin, with pink mycelium for *Lentzea* sp. CC55 and yellow mycelium for *Actinokineospora* sp*.* PR83. Using Illumina sequencing, we determined general genome assembly features: *Lentzea* sp. CC55 encodes 8.27 Mb (70.5 mol% G+C, 101 contigs, N_50_ 145251) and *Actinokineospora* sp. PR83 encodes 7.64 Mb (72.7 mol% G+C, 182 contigs, N_50_ 77654) genomes (Table 1). The genome of *Lentzea* sp. CC55 encodes a total of 7675 proteins, 9 rRNA genes and 69 tRNA genes, while *Actinokineospora* sp. PR83 encodes a total of 6565 proteins, 13 rRNA genes and 50 tRNA genes. Using the Microbial Genome Atlas platform (MiGA version 1.1.2.2) [52], we determined that *Lentzea* sp. CC55 shares 96.90 % average nucleotide identity (ANI) with *Lentzea jiangxiensis* CGMCC 4.6609 [53], and *Actinokineospora* sp. PR83 shares 95.9 % ANI with *Actinokineospora spheciospongiae* EG49 [54]. *Lentzea* sp. CC55 and *Actinokineospora* sp. PR83 genome sequences produced in this study have been deposited at GenBank/ENA/DDBJ under the accession numbers JAJCXE000000000 and JAJCXD000000000, respectively.

Using the BiNI scores, we ranked according to biosynthetic novelty all the *Lentze* a and *Actinokineospora* strains reported in genomic databases. The strain *Lentzea* sp. CC55, isolated from sediments of an oligotrophic environment, has the highest degree of biosynthetic novelty (BiNI=1 008.6) among all the *Lentzea* strains in our database. Another *Lentzea* strain with potential of encoding novel BGCs is *Lentzea flava* (BiNI=777.8), isolated from soil, Acharya Nagarjuna, India [55], and *Lentzea nigeriaca* (BiNI=742.5) isolated from desert soil in Nigeria [56] (Table S1). Regarding the strains of *Actinokineospora *, based on the BiNI, we found that the strains *A. fastidiosa* isolated from desert soil samples in Egypt [57], '*Actinokineospora xionganensis'* isolated from lakeside soil samples in China [58], and *Actinokineospora* sp. PR83 isolated from oligotrophic environments in Mexico had the highest BiNI values (1097.2, 1050.5 and 1049.3, respectively). In contrast, strains *Actinokineospora pegani* isolated from root of *Peganum harmala*, China [59], and *Actinokineospora mzabensis* isolated from Saharan desert soil [60], Southern Algeria, had the lowest BiNI scores (81.3 and 117.7, respectively) (Table S1).

To support the idea that nutrient-limiting environments harbour micro-organisms with novel biosynthetic potential, we compared the genomes of a group of free-living *Streptomyces* sp. 8s0 isolated in the Cuatro Cienegas Basin, seven endophytic *Micromonospora* (BiNI=284.3), seven endophytic *Streptomyces* with a group of reference *Streptomyces* genomes (*Streptomyces_xiamenensis*_strain 318, *Streptomyces_rapamycinicus* strain NRRL 5491 and Streptomyces*_coelicolor* strain A32) (Table S1). We chose the *Streptomyces* genomes of different length to avoid a possible bias caused by the number of BGCs. According to the BiNI score, isolates from nutrient-limited environments encode BGCs of higher novelty as the groups of *Streptomyces* from Cuatro Cienegas Basin and the endophytic *Streptomyces* showed a BiNI score of 525.8 and 376, respectively. The three reference *Streptomyces* genomes used showed a BiNI score of 144.5 (Fig. 1a).

### Phylogenomic analysis of *Lentzea* sp. CC55 and *Actinokineospora* sp. PR83 isolates

**Fig. 2.**
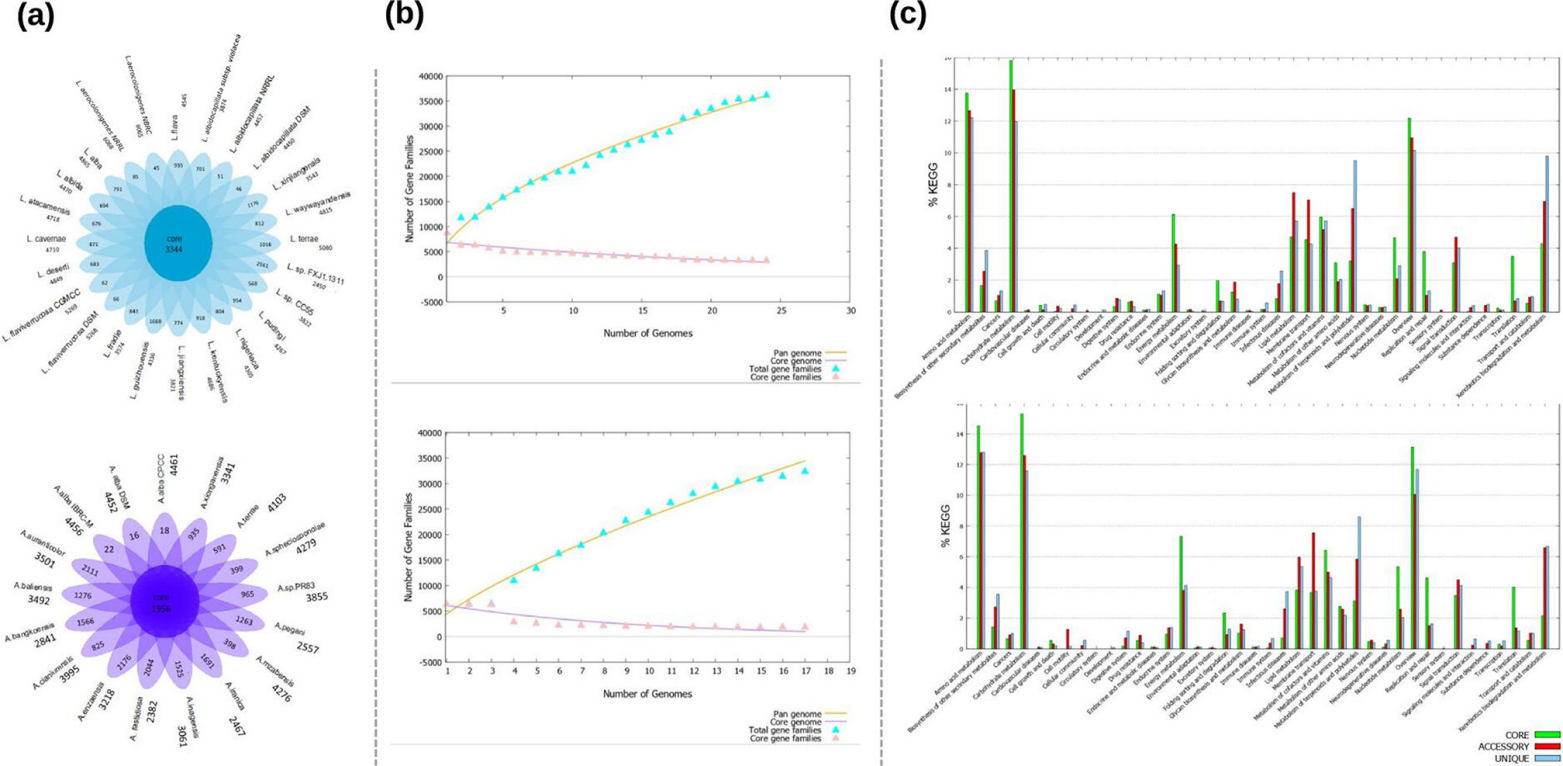
Pan-genome analysis for *Lentzea* and *Actinokineospora* genomes. (a) Flower diagrams representing the core, unique and accessory genes for each genus. The numbers underneath the names represent the number of accessory genes. (**b)** Core-/pan-genome plot over 500 iterations using the bpga analysis tool. The pan-genome is open. (**c)** KEGG functional analysis of the pan-genome. The graph shows the predicted function of proteins encoded by core (green), accessory (red) and unique (blue) genes of the pan-genome using KEGG identification.

To provide insights into the genetic diversity that accounts for adaptation capabilities and population structure, we analysed *Lentzea* and *Actinokineospora* pan-genomes using the bpga tool. Our analysis includes all of the genomes reported for both *Lentzea* and *Actinokineospora* up to September 2021 (see Table 1). The pan-genome of *Lentzea* is estimated at 33 321 genes, including a core genome of 3344 genes, a mean accessory genome of 4513 genes and a total of 17 800 unique genes (Fig. 2a). *Lentzea aerocolonigenes* (strains NBRC 131195 and NRRL B-3298), *Lentzea albidocapillata* (strains DSM 44073 and NRRL B-24057) and *Lentzea flaviverrucosa* (strains CGMCC 4.578 and DSM 44664) encode the smallest number (range 45–85) of unique genes within the pan-genome. In contrast, *Lentzea terrae* DSM 44260 (1 016 genes), *Lentzea xinjiangensis* CGMCC 4.3525 (1 176 genes), *Lentzea guizhouensis* DHS C013 (1 668 genes) and *Lentzea* sp. FXJ1.1311 (2561 genes) encode an almost 20 times larger number of unique genes (Table S2). Our strain, *Lentzea* sp. CC55, encodes 568 unique genes. The pan-genome of *Actinokineospora* is estimated at 32 010 genes, including a core genome of 1956 genes, a mean accessory genome of 3573 genes and a total of 17 821 unique genes (Fig. 2a). *Actinokineospora alba* strains DSM 45114 (16 genes), CPCC 201030 (18 genes) and IBRC-M 10655 (22 genes) present a small number of unique genes compared to *A. fastidiosa* JCM 3276, *Actinokineospora auranticolor* YU 961–1 and *Actinokineospora enzanensis* DSM 44649, which encode much larger numbers of unique genes (2044, 2111 and 2176 genes, respectively) (Table S2). Our strain, *Actinokineospora* sp. PR83, encodes 965 unique genes.

Inspection of the core–pan plots indicated that both pan-genomes are open. This highlights the strong requirement to increase efforts for isolation in these genera (Fig. 2b). Functional annotation using the KEGG (Kyoto Encyclopedia of Genes and Genomes) database for the pan-genomes indicates that most of the core, accessory and unique genes are related to central metabolic pathways (Fig. S1). According to the KEGG functional classification, 13–15 % of the pan-genome for *Lentzea* (core 20, unique 31, accessory 21) and *Actinokineospora* (core 28, unique 32, accessory 29) belong to amino acid metabolism and carbohydrate metabolism and, overall, the genes involved in these functions would represent the major contributors to the genetic diversity observed in each genus. As expected, for both genera, most of the unique genes pertain to the specialized metabolism of terpenoids and PKs, as well as the biodegradation of xenobiotics metabolism (Fig. 2c).

**Fig. 3.**
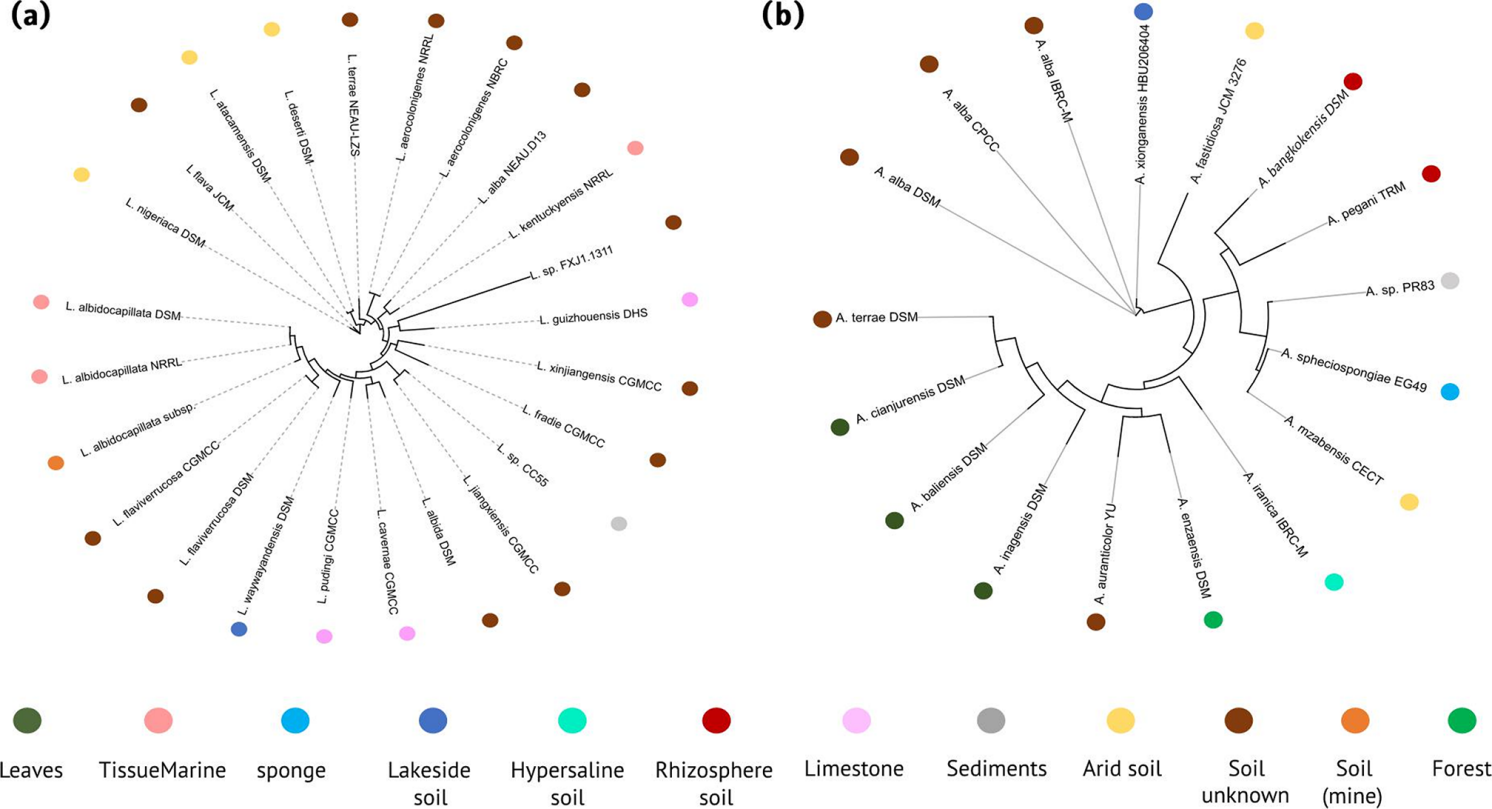
Phylogeny trees based on the pan-matrix from bpga analysis for the *Lentzea* (**a**) and *Actinokineospora* (**b**) strains. The alignment was performed with muscle v.3.8.31. The characteristics of isolation for each genome are visualized by colour.

To infer the phylogenetic relationships among members of both *Lentzea* and *Actinokineospora *, we reconstructed a phylogenetic tree using the core genome derived from the pan-genome analysis. For the *Lentzea* phylogenetic tree (Fig. 3a), 24 strains were grouped into two clades, with the exception of *L. flava* (soil, Acharya Nagarjuna, India) and *L. nigeriaca* (arid soil from Nigeria). One clade contained two *Lentzea* strains isolated from arid soils (Atacama Desert of Northern Chile) [61], and the second clade contained 20 strains isolated from different soil sources (Table 1). The strains *Lentzea deserti* and *Lentzea atacamensis* (isolated from arid soils); *L. albidocapillata* DSM and NRRL (isolated from samples of tissue); *L. aerocolonigenes* NRRL and NRBC (isolated from soil, Japan); *L. xinjiangensis* and *Lentzea fradiae* (soil, China); and *L. flaviverrucosa* DSM and CGMCC (soil, Shanxi Province, China) were subgrouped according to the place of isolation. Our strain, *Lentzea* sp. CC55, was closely related to *L. jiangxiensis* strain CGMCC 4.6609 (soil isolate from Jiangxi Province, South-East China) [50].

For the phylogenetic analysis of *Actinokineospora* (Fig. 3b), 17 strains were grouped into two clades, with the exception of the *A. alba* strains (soil, Xinjiang Province, China) [62], '*A. xionganensis'* (lakeside soil Xiongan, China) [58] and *A. fastidiosa* (arid soil, Egypt) [57]. In the first clade, the strains *Actinokineospora bangkokensis* DSM [63] and *A. pegani* TRM (both from rhizosphere soils) [59] are grouped together, while our strain, *Actinokineospora* sp. PR83, *A. spheciospongiae* (marine sponge) [54] and *A. mzabensis* CECT (desert soil) [60] form another subclade. *A. enzanensis* DSM (soil, Yamanashi Japan) [64] and *A. auranticolor* YU [65] form a monophyletic group with three strains isolated from tree leaves as well as *Actinokineospora terrae* (soil, Japan) [66]. Only the strains of *A. alba* isolated from soil, China; *A. bangkokensis* and *A. pegani* isolated from rhizospheric soil; and *Actinokineospora cianjurensis *, *Actinokineospora baliensis* and *Actinokineospora inagensis* isolated from leaves were subgrouped according to the ecological context.

### *Lentzea* sp. CC55 presents a higher number of BGCs grouped as singletons compared to other *Lentzea* strains

**Fig. 4.**
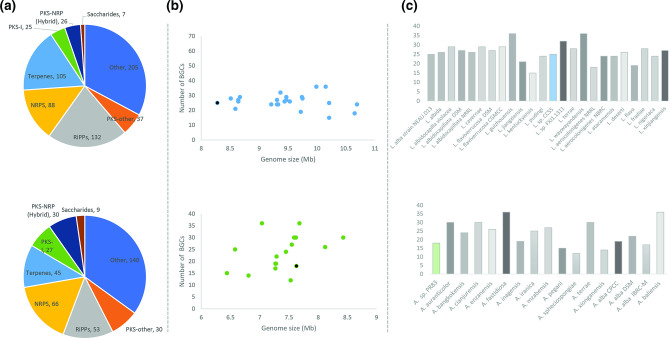
Distribution of BGCs according to antiSMASH identity. (a) Circular representation of BGC family distribution for *Lentzea* (circle at the top) and *Actinokineospora* (circle at the bottom). (**b)** Genome length versus number of clusters for *Lentzea* (blue; graph at the top) and *Actinokineospora* (green; graph at the bottom). Black circles represent *Lentzea* sp. CC55 and *Actinokineospora* sp. PR83, respectively. (**c)** Histogram for number of clusters by strain for *Lentzea* (blue; graph at the top) and *Actinokineospora* (green; graph at the bottom).

Using antiSMASH (version 6.0) [46], we identified 32 BGCs (25 complete) for *Lentzea* sp. CC55, including 6 NRPSs, 7 PKSs, 4 terpenes, 4 RiPPs and 4 classified as ‘other’. A total of seven BGCs showed medium-to-high (50–100 %) homology with previously identified clusters (two geosmins, 2-methylisoborneol, coelichelin, Ery-9 and methoxyhydroquinones). Fourteen clusters were identified with low homology (1–36 % identity) and four BGCs presented no match in the database (see Table S3). We analysed the biosynthetic potential (number of complete BGCs) for all the genomes of *Lentzea* publicly available, taking into account genome size and quality of the assembly (number of contigs). We identified a total of 625 BGCs in all of the genomes (mean=26 BGCs per genome), where 14 % were classified as non-ribosomal peptides, 17 % as terpenes, 21 % as RiPPs, 10 % as PK synthases, 4 % as hybrid PKS-NRP and 2 % as saccharides. A total of 205 BGCs either belonged to other chemical families or had no defined classification according to available databases (Fig. 4a). We observed an even number of BGCs (mean=25±10) (Fig. 4b). The highest numbers of BGCs in *Lentzea* were found in *Lentzea waywayandensis* (36 BGCs, 10.15 Mb, 39 contigs), *L. guizhouensis* (36 BGCs, 9.99 Mb, 1 contig) and *Lentzea* sp. FXJ1.1311 (32 BGCs, 9.37 Mb, 120 contigs), while *Lentzea* sp. CC55 genome encodes 25 BGCs in a genome of 8.28 Mb assembled in 101 contigs. The strain with the lowest number of BGCs was *Lentzea kentuckyensis* (15 BGCs, 10.21 Mb, 317 contigs) (Fig. 4c).

**Fig. 5.**
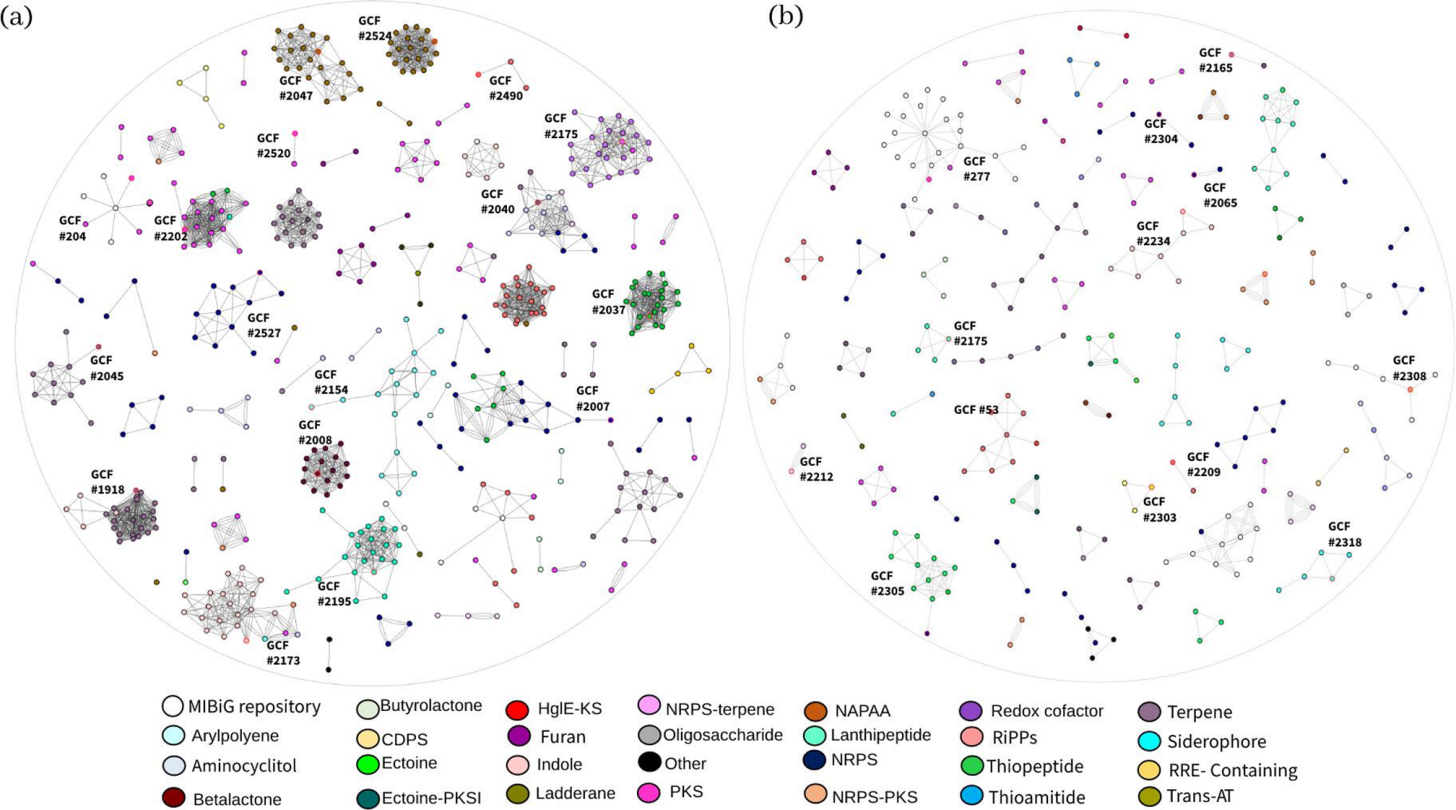
BGC network showing chemical diversity for*Lentzea* (**a**) and *Actinokineospora* (**b**) strains. Each colour node corresponds to a BGC associated with a chemical family. BGCs with red circular borders are those of . *Lentzea* sp. CC55 and *Actinokineospora* sp. PR83. Singletons were not included in the sequence similarity networks due to having a distance lower than 0.3. HglE-KS, Heterocyst glycolipid synthase-like; NAPAA, non-alpha poly-amino acids (such as e-polylysine).

In order to study the repertoire of specialized metabolism-related clusters of all of the *Lentzea* strains, we constructed BGC sequence similarity networks including the 1802 BGCs reported in the MIBiG database (September 2021). We used a raw distance cut-off of 0.3 to observe the BGC relationships among strains, highlighting the principal gene cluster families (GCFs) related to *Lentzea* sp. CC55 (Fig. 5a, Table S3). Most of the GCFs conserved by the *Lentzea* strains were related to the category other, including siderophores, redox cofactors and indole, followed by RiPPs such as non-alpha poly-amino acids like e-Polylysin (NAPPA), lanthipeptides and thiopeptides; terpenes; NRPS and PKS. Based on this analysis, we observed that the BGCs from *L. flaviverrucosa* DSM and *L. flaviverrucosa* CGMCC (isolated from soils from Shanxi Province, China) [67] are conserved among all members of the genus *Lentzea *. In contrast*, Lentzea* sp. FXJ1.1311 isolated from soil in Tibet, China [68], and *L. guizhouensis* isolated from limestone [69], Guizhou Province in South-Western China, were the strains with the highest number of unique BGCs (25/32 and 20/36, respectively). On comparison of the BGCs to the MIBiG database, we found a match for only 5/280 GCFs (GCF 2362, GCF 79, GCF 204, GCF 1278 and GCF 1951). We assigned 17 out of 25 BGCs from *Lentzea* sp. CC55 to a known GCF, while 8 BGCs were identified as singletons (Table S3).

### *Actinokineospora* sp. PR83 encodes a unique subset of BGCs

*Actinokineospora* sp. PR83 encodes 18 complete BGCs, including 6 PKSs, 3 NRPSs, 1 terpene, 2 RiPPs and 6 classified as other. Amongst all BGCs, only two showed medium-to-high homology with previously characterized NPs, including PKs similar to streptovaricin, and JBIR-76/JBIR-77. Twelve BGCs that showed 1–44 % identity included hybrids (such as kosinostatin, kalimantacin A and oxalomycin B), PKs (saquayamycin A, chlorothricin, macrotetrolide and dynemicin), non-ribosomal peptides similar to atratumycin and gobichelin A, the saccharides apramycin, methylenomycin A, and cetoniacytone A, classified as other. A total of four BGCs presented no match in the database (Table S4). When we consider the entire genus *Actinokineospora *, we identified a total of 400 BGCs (23.5 BGCs per genome), of which 17 % were classified as non-ribosomal peptides, 14 % were PK synthases, 13 % were RiPPs, 11 % were terpenes, 8 % hybrid PKS-NRPS, 2 % were saccharides and 35 % were not classified into a principal chemical family (Fig. 4a). In contrast to the genus *Lentzea *, the number of clusters does not present a linear relationship to genome size (Fig. 4b). The genomes with the highest number of BGCs were *A. fastidiosa* (36 BGCs, 7.04 Mb, 30 contigs), *A. baliensis* (36 BGCs, 7.68 Mb, 1 contig), *A. auranticolor* (30 BGCs, 8.43 Mb, 61 contigs) and *A. terrae* (30 BGCs, 7.59 Mb, 41 contigs), while the genome with the lowest number of BGCs was *A. spheciospongiae* (12 BGCs, 7.53 Mb, 263 contigs) (Fig. 4c).

When we compared the biosynthetic space of all of the *Actinokineospora* strains using a homology network, we found that most of the GCFs were related to NRPS, followed by the category other, including siderophores, ectoines, indole, terpenes and RiPPs. The BGCs from *Actinokineospora iranica* (isolated from soil, Inche-Broun hypersaline wetland, North Iran) [70] and *A. alba* IBRC-M (isolated from soil, Xinjiang Province, China) [62] are part of the ‘biosynthetic core genome’ for the genus *Actinokineospora *. In contrast, *A. fastidiosa* and *A. auranticolor* were the strains with the highest number of singletons. When comparing the BGCs to the MIBiG database, we found a match for only nine GCFs (Fig. 5b, Table S4). We assigned 14 out of 18 BGCs from *Actinokineospora* sp. PR83 to a known GCF, including BGCs similar to oxalomycin B, NRPS, saquayamycin A, indole, atratumycin, dynemicin A, RiPP-like, apramycin, gobichelin A, macrotetrolide and streptovaricin.

### *Lentzea* sp. CC55 and *Actinokineospora* sp. PR83 show antimicrobial activity against Gram-positive pathogens and cytotoxicity

After studying the *in silico* biosynthetic potential (number of BGCs) of *Lentzea* sp. CC55 and *Actinokineospora* sp. PR83, we used the OSMAC strategy to screen BGCs with antimicrobial or anticancer activity. In order to cover different N and C sources, we analysed extracts from eight different media (ISP-2, ISP-4, R5, Czapek, oatmeal, BS, BG and M1), from both liquid and solid cultures. We correlated the results obtained from the antimicrobial (against *B. subtilis *, *Staphylococcus aureus *, *E. coli* and *P. aeruginosa *) and anticancer (NCIH460 cell line) activity assays to their corresponding UHPLC (ultra-HPLC) chromatograms. This approach allowed us to identify potential signals in the chromatograms for use as leads for the purification of molecules responsible for the bioactivity.

**Fig. 6.**
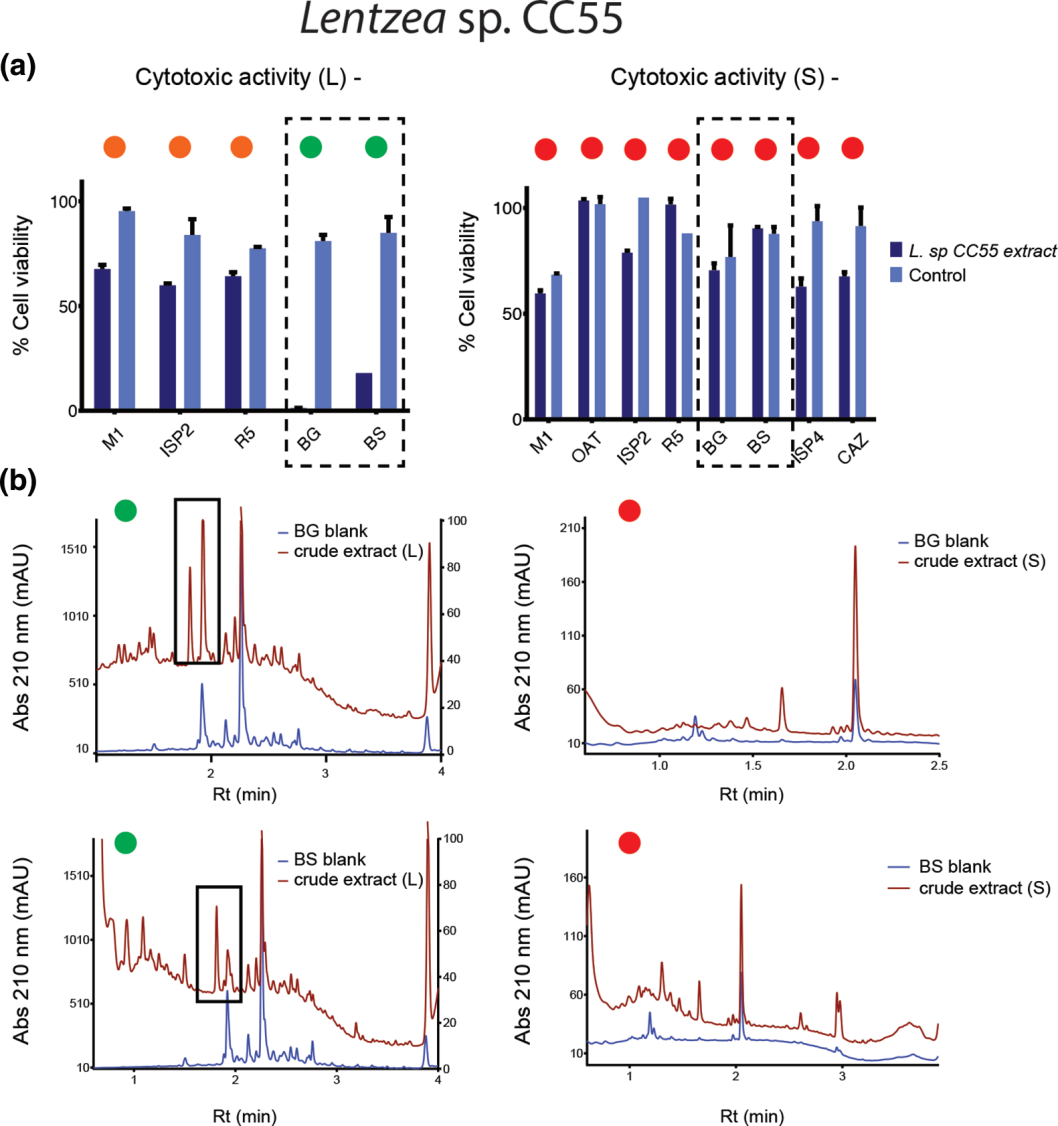
Cytotoxicity assays and chromatography results from liquid and solid culture media of *Lentzea* sp. CC55. (a) Dashed line boxes highlight the highest cytotoxic effect on NCIH460 cells related to a reduction in cell viability. Null, moderate and high cytotoxic effect can be observed as red, orange and green circles at the top of the bioactivities. (**b)** Comparison between chromatograms from extracts from liquid BG and BS media that presented cytotoxicity and their counterparts from solid media with no bioactivity. Presence (green) or absence (red) of peaks in chromatograms related to bioactivities are shown at the top with green and red circles, respectively. Rt, retention time. mAU, milli-absorbance unit

**Fig. 7.**
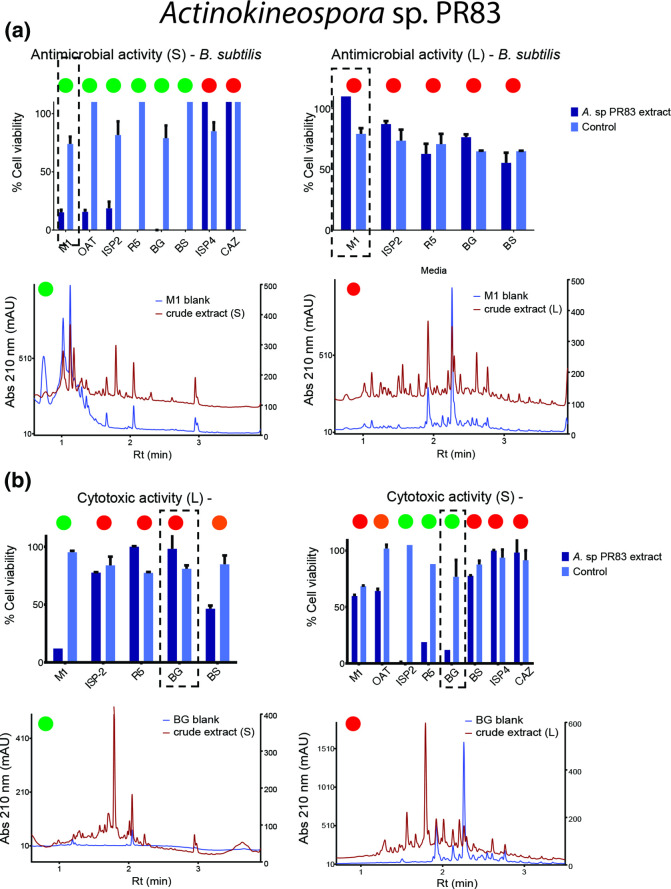
Bioactivity assays and chromatography results from liquid and solid culture media of *Actinokineospora* sp. PR83. (a) Antimicrobial activity against *B. subtilis *. (**b)** Cytotoxic effect on NCIH460 cells. For a and b, dashed line boxes highlight the highest antimicrobial or cytotoxic effect related to a reduction in cell viability. Null, moderate and high antimicrobial or cytotoxic effect can be observed as red, orange and green circles at the top of the bioactivities, respectively. Presence (green) or absence (red) of peaks in chromatograms related to bioactivities are shown at the top with green and red circles, respectively. Rt, retention time. mAU, milli-absorbance unit.

*Lentzea* sp. CC55 culture extracts from BG and BS media showed cytotoxic activity only in liquid cultures (Fig. 6a). We used the chromatograms obtained from BG and BS to identify a peak (1.8 min retention time) that might be responsible for the observed activity, since such signals are not observed in BG and BS solid media chromatograms (Fig. 6b). Regarding *Actinokineospora* sp. PR83 extracts, we observed antimicrobial activity against Gram-positive pathogens such as *B. subtilis* for all the solid media tested (<10 % cell viability). In contrast, the extracts of liquid media did not present a significant antimicrobial effect on pathogen cell viability. Positive chromatograms were correlated with the signals found in negative extracts for peak identification. For example, the bioactivity of extracts from solid M1 medium was related to one peak closest to 0.5 min retention time, while this signal is absent in liquid M1 extracts (Fig. 7a). However, *Actinokineospora* sp. PR83 extracts also showed a cytotoxic effect on the carcinoma cell line in M1 and BS liquid extracts (M1, <10 % cell viability; BS, <50 % cell viability) and in ISP-2, R5 and BG solid extracts (ISP-2, R5, BG, <10 % cell viability). Given that we observed two signals with more than one significant bioactivity, it is difficult to differentiate chromatography signals for each bioactivity (Fig. 7b).

## Discussion

The first milestone of the microbial-based drug discovery process is the establishment of the correlation strain–metabolite–bioactivity. During this process, re-discovery of known metabolites is inevitable, since isolation methods using conventional culture media have been over utilized. A plausible solution to this problem is the development of protocols for the systematic prioritization of strains based on the biosynthetic novelty. For instance, the isolation of uncommon/underisolated actinobacteria, also called rare actinobacteria, has created new opportunities for the discovery of new metabolites [13–15, 17–19]. In this study, we defined the BiNI to provide a quantitative framework for classification of strains according to their biosynthetic novelty. We demonstrated that the BiNI score can be used to compare biosynthetic novelty within a subset of genomes and to draw comparisons between ecological niches and taxonomic groups.

At the level of taxonomic genera, *Streptosporangium *, *Actinokineospora* and *Saccharothrix* showed the highest novelty index, while *Thermobifida* and *Saccharomonospora* showed the lowest BiNIs. When comparing strains of the same genus, our results suggest that bacteria inhabiting highly oligotrophic environments (e.g. desert soils) encode a novel subset of BGCs. We observed that for the *Actinokineospora* and *Lentzea* genomes tested, the strains with highest BiNI values were isolated from the Cuatro Cienegas Basin and desert soil samples from Egypt (* A. fastidiosa *). In addition, when we compared *Streptomyces* strains from different environments, including endophytic strains, we observed that environments with low levels of nutrients harbour strains with higher biosynthetic novelty.

*Lentzea* sp. CC55 and *Actinokineospora* sp. PR83 were isolated from the Cuatro Cienegas Basin in Mexico. The soils of the Cuatro Cienegas Basin are characterized by a extremely unbalanced N:P (157 : 1) soil stoichiometry, and rich sulfur, magnesium and calcium sulfate concentrations [71]. It has previously been shown that these conditions conserve a great diversity of endemic microbes with novel biosynthetic potential [32, 72]. For instance, our research group has previously described new diversity in desferrioxamine siderophores (propionyl-ferrioxamine, *m*/*z*=628.17; aryl-ferrioxamine, *m*/*z*=690.26) in species of *Microbacterium *, *Micrococcus* and *Streptomyces* [33].

We performed a pan-genomic analysis using publicly available genomes and found a high number of unique genes for both genera (~17 800 per each genus for *Lentzea* and *Actinokineospora *) compared to other rare actinobacteria isolated from extreme polar environments [73]. We found that novel genes are mostly distributed in specialized metabolite-related genes. The rarefaction curves obtained indicate that more genomes are required in order to cover the entire genetic space within these taxonomic genera. The phylogenetic analysis derived from the pan-genomes found no correlation between the ecological contexts of the *Lentzea* and *Actinokineospora* strains.

Genomic analysis using GCFs is useful to classify BGCs according to their functional families and biosynthetic novelty [74, 75]. Initial inspection of the GCFs from our strains showed a high number of singletons, which represent potential novel BGCs. While a direct correlation between genome size and number of BGC is expected [76], we observed a correlation between the taxonomic genus and number of clusters. Expression of the biosynthetic potential of environmental isolates in the laboratory is limited by the capacity to artificially reproduce culture conditions that resemble those of the isolation site. In our study, we evaluated the capacity of our strains to express BGCs with antimicrobial and cytotoxic activity using the OSMAC strategy. We correlated the chromatograms obtained from different media settings (liquid or solid cultures) and media formulations (different C and N sources). The bioactivity test allowed us to identify the most suitable culture conditions for directing purification of the NPs with relevant bioactivity. As expected, we found that solid media favours a higher expression of BGCs responsible for antimicrobial and cytotoxicity activity compared to liquid cultures [77–81]. Solid substrates maintain the membrane skeleton, allowing the hydrophobicity for aerial mycelium growth and, consequently, metabolite production [82]. However, at large-scale, solid media cultures are not feasible for metabolite characterization (chemical composition determination) since the production requires high-yield processes performed in a bioreactor. The OSMAC approach combined with bioactivity assays helped us to correlate chromatography signals to bioactivity. We consider that this method can be used as the primary experimental approach for the chemical characterization of novel compounds. However, it is important to note that this method can only be used with extracts that show a single positive bioactive chromatography signal.

Overall, our study provides a full genomic and phenotypic characterization of two rare actinobacteria from the genera *Lentzea* and *Actinokineospora *. Our genomic analysis also provides a metric for quickly prioritizing strains according to the novelty of their BGCs. While the biosynthetic novelty of the strains we analysed from the Cuatro Cienegas Basin was not as high as that of other genera of rare actinobacteria, our strains show high novelty when comparing isolates from other ecological niches. This highlights the potential of exploring highly oligotrophic environments in the search for novel NPs. More importantly, we consider that our study contributes to the development of strategies for the rapid prioritization of strains isolated from different ecological contexts.

## Supplementary Data

Supplementary material 1
