# Supplementary material for: Biosynthetic novelty index reveals the metabolic potential of rare actinobacteria isolated from highly oligotrophic sediments
Source: Microb Genom. 2023 Jan 20;9(1):mgen000921. doi: 10.1099/mgen.0.000921 (PMC9973853; doi:10.1099/mgen.0.000921)

Supplementary table 1. Genomic characteristics of rare taxonomic genera and endophytes used for biosynthetic novelty calculation.

Accesion Number	Genome Name	Contigs	Genome Length	%GC	BiNI
GCF_900115955.1	*Lentzea waywayandensis* strain DSM 44232	39	10153412	68.9	334.4
GCF_011067745.1	*Lentzea alba* strain NEAU.D13	42	10211123	68.7	267.5
GCF_900100275.1	*Lentzea albidocapillata* subsp. violacea DSM 44796	58	8671075	69	368.7
GCF_900110985.1	*Lentzea flaviverrucosa* strain CGMCC 4.578	34	9473544	69.2	188.1
GCF_900104175.1	*Lentzea* jiangxiensis strain CGMCC 4.6609	62	8591279	70.2	257.6
GCF_900111005.1	*Lentzea albida* strain DSM 44437	43	9441135	70.2	439.3
GCF_900176525.1	*Lentzea albidocapillata* strain DSM 44073	43	8639486	68.7	369.3
GCF_003350445.1	*Lentzea flaviverrucosa* strain DSM 44664	38	9468454	69.2	202
GCF_003265345.1	*Lentzea terrae* strain NEAU-LZS 42	145	10581732	68.6	254.9
GCF_000719115.1	*Lentzea albidocapillata* strain NRRL B-24057	127	8642480	68.7	334.6
GCF_014646255.1	*Lentzea pudingi* strain CGMCC 4.7319	63	9209394	69.1	774.2
GCF_002150765.1	*Lentzea kentuckyensis* strain NRRL B-24416	317	10210611	68.8	350.9
GCF_007845675.1	*Lentzea sp*. strain FXJ1.1311	120	9370857	69.5	565.8
GCF_001701025.1	*Lentzea guizhouensis* strain DHS C013	1	9997872	70	348.4
GCF_014653695.1	*Lentzea cavernae* strain CGMCC 4.7367	43	9738650	69.6	694
GCF_000974445.1	*Lentzea aerocolonigenes* NBRC 13195	61	10698154	69	277
GCF_003269295.1	*Lentzea atacamensis* DSM 45479	38	9306230	68.9	365
GCF_016907955.1	*Lentzea nigeriaca* DSM 45680	42	9332327	68.4	742.5
GCF_900100955.1	*Lentzea fradiae* CGMCC 4.3506	47	8508028	70.5	382.5
GCF_003148865.1	*Lentzea deserti* DSM 45480	50	9529573	68.8	423.3
GCF_900110955.1	*Lentzea xinjiangensis* CGMCC 4.3525	61	8684108	68.9	378.4
GCF_014648475.1	*Lentzea flava* JCM 3296	190	9721257	69	777.8
GCF_000719205.1	*Lentzea aerocolonigenes* NRRL B-3298	204	10648728	69	297
GCF_021056325.1	*Lentzea sp*. CC55	101	8269994	70.5	1008.6
GCF_000374445.1	*Actinokineospora enzanensis* strain DSM 44649	95	8119858	70.8	290.5
GCF_014323725.1	*Actinokineospora xionganensis* HBU206404	70	6813329	69.6	1050.5
GCF_900111175.1	*Actinokineospora terrae* strain DSM 44260	41	7588185	71	254.9
GCF_001940455.1	*Actinokineospora bangkokensis* strain DSM 44EHW	78	7453713	74	267.9
GCF_003182415.1	*Actinokineospora mzabensis* strain CECT 8578	32	7546603	72.8	117.7
GCF_002934265.1	*Actinokineospora auranticolor* strain YU 961-1	61	8431890	71.8	389
GCF_000482865.1	*Actinokineospora inagensis* strain DSM 44258	120	7278759	70.2	244.5
GCF_000564855.1	*Actinokineospora spheciospongiae* strain EG49	263	7529476	72.8	260.1
GCF_003663795.1	*Actinokineospora cianjurensis* strain DSM 45657	19	7615874	70.9	173.4
GCF_009745975.1	*Actinokineospora pegani* strain TRM65233	137	6439512	72.6	81.3
GCF_014648415.1	*Actinokineospora fastidiosa* strain JCM 3276	30	7042850	72.5	1097.2
GCF_004362515.1	*Actinokineospora alba* DSM 45114	1	7288615	69.7	409.8
GCF_016907695.1	*Actinokineospora baliensis* DSM 45656	1	7681387	70.6	971
GCF_900101685.1	*Actinokineospora iranica* IBRC-M 10403	44	6578763	70.8	205.3
GCF_900099755.1	*Actinokineospora alba* CPCC 201030	35	7273063	69.7	471.7
GCF_900104505.1	*Actinokineospora alba* IBRC-M 10655	34	7268999	69.7	342.2
GCF_021056305.1	*Actinokineospora sp.* PR83	182	7639714	72.7	1049.3
GCF_900188345.1	*Streptosporangium subroseum* CGMCC 4.2132	180	10782574	68.8	195.3
GCF_014636435.1	*Streptosporangium jomthongense* CGMCC 1.11045	55	4127297	55.8	0
GCF_013409935.1	*Streptosporangium sandarakinum* DSM 45763	4	7647619	72.5	961.3
GCF_014203715.1	*Streptosporangium saharense* CECT 8840	26	8496112	70.3	1120.5
GCF_014191075.1	*Streptosporangium becharense* CECT 8961	37	7762257	71.4	764.5
GCF_014204985.1	*Streptosporangium becharense* DSM 46887	2	7824014	71.3	877.8
GCF_000725815.1	*Streptosporangium roseum* NRRL B-2638	308	10168636	70.6	230.6
GCF_000721075.1	*Streptosporangium amethystogenes* NRRL B-2639	229	9441077	69.4	211.4
GCF_003203775.1	*Streptosporangium* sp. 'caverna'	1	11342172	68.9	384
GCF_900113865.1	*Streptosporangium canum* CGMCC 4.2126	91	9273799	70.7	229.6
GCF_014648055.1	*Streptosporangium pseudovulgare* JCM 3115	73	8586701	72.5	958.4
GCF_014654615.1	*Streptosporangium violaceochromogenes* JCM 3281	115	6265008	71.0	730.9
GCF_014203795.1	*Streptosporangium album* DSM 43023	14	9022456	69.4	745
GCF_000024865.1	*Streptosporangium roseum* DSM 43021	2	10369518	70.9	267.9
GCF_000401915.1	*Thermobifida fusca* TM51	88	3599272	47.6	339.3
GCA_001313945.1	*Thermobifida fusca* NBRC 14071 = JCM 3263	82	3643897	67.5	0
GCF_015034585.1	*Thermobifida fusca* UPMC 901	1	3760927	67.4	175.5

GCF_002939325.1	*Thermobifida fusca* GAFM-TF-ZOO	129	3638186	67.5	0
GCA_003242745.1	*Thermobifida fusca* ZC4RG21	204	3609530	67.6	0
GCF_902797715.1	*Thermobifida fusca* RUG14297	190	3506582	67.7	0
GCF_000012405.1	*Thermobifida fusca* YX	1	3642249	67.5	169.7
GCF_001660385.1	*Thermobifida halotolerans* YIM 90462	74	5453353	70.8	265.3
GCF_003574835.2	*Thermobifida halotolerans* DSM 44931	314	5217717	70.9	314.3
GCF_001517975.1	*Thermobifida cellulosilytica* TB100	19	4430365	70.4	288.3
GCF_009498035.1	*Saccharothrix syringae* NRRL B-16468	1	10929570	73.4	108.4
GCF_003752005.1	*Saccharothrix texasensis* DSM 44231	1	9178199	72.2	147.1
GCF_014203665.1	*Saccharothrix tamanrassetensis* CECT 8640	31	8053586	71.4	1122.1
GCF_014205015.1	*Saccharothrix ecbatanensis* DSM 45486	1	9689237	70.1	1199.7
GCF_000328705.1	*Saccharothrix espanaensis* DSM 44229	1	9360653	72.1	252.6
GCF_003014735.1	*Saccharothrix carnea* CGMCC 4.7097	40	8918169	71.4	157.3
GCF_001280085.1	*Saccharothrix* sp. NRRL B-16348	279	10896489	71.3	209.2
GCF_000716595.1	*Saccharothrix* sp. NRRL B-16314	273	8934493	71.1	275.2
GCF_001905065.1	*Saccharothrix* sp. CB00851	66	9304003	71.2	210.6
GCF_001984175.1	*Saccharothrix* sp. ALI-22-I strain ALI-22-I	69	10300059	70.1	345.2
GCF_003634935.1	*Saccharothrix australiensis* DSM 43800	3	7861373	73.4	233.8
GCF_009769385.1	*Saccharothrix* sp. BMP B8144	162	10832261	70.3	290.1
GCF_016695385.1	*Saccharothrix* sp. 6-C	1	9045220	72.3	272.2
GCF_014648515.1	*Saccharothrix coeruleofusca* JCM 3313	61	7692265	72.6	1084
GCF_014203755.1	*Saccharothrix violaceirubra* DSM 45084	1	7354386	71.2	994.5
GCF_003634995.1	*Saccharothrix variisporea* DSM 43911	1	9408895	71.6	339.8
GCF_006716745.1	*Saccharothrix saharensis* DSM 45456	1	8924967	72.2	211.5
GCF_000236985.1	*Saccharomonospora azurea* SZMC 14600	216	4973727	70.3	0
GCF_011761185.1	*Saccharomonospora amisosensis* DSM 45685	3	5534940	68.5	555.5
GCF_000527075.1	*Saccharomonospora* sp. CNQ490	25	4941689	71.1	244.5
GCF_015910535.1	*Saccharomonospora sp. NB11*	29	4299407	69.8	1148.4
GCF_000787535.1	*Saccharomonospora viridis* JCM 3315	13	4304348	67.3	0
GCF_900115515.1	*Saccharomonospora viridis* ATCC 33517	12	4308214	67.3	0
GCF_001942305.1	*Saccharomonospora* sp. CUA-673	85	5421117	69.9	666.7
GCF_004519465.1	*Saccharomonospora* sp. 31sw	1	4713652	69.0	0
GCF_000023865.1	*Saccharomonospora viridis* DSM 43017	1	4308349	67.3	0
GCF_002077655.1	*Saccharomonospora piscinae* strain 06168H-1	11	4897614	71.0	79.6
GCF_000231055.2	*Saccharomonospora azurea* NA-128	93	4662072	64.1	238
GCF_000244975.1	*Saccharomonospora cyanea* NA-134	5	5407901	69.7	254.6
GCF_000244955.1	*Saccharomonospora marina* XMU15	8	5964893	68.9	182.4
GCF_000258175.1	*Saccharomonospora xinjiangensis* XJ-54	16	4774606	68.9	156.3
GCF_000243395.2	*Saccharomonospora glauca* K62	11	4563208	69.1	91.6
GCA_000374705.1	*Salinispora pacifica* CNS860	72	5351008	69.6	210.4
GCA_000383995.1	*Salinispora pacifica* DSM 45543	2	5458189	69.6	171.8
GCA_000378845.1	*Salinispora pacifica* DSM 45544	29	5462252	69.9	374.3
GCA_000374745.1	*Salinispora pacifica* CNS996	119	5658754	69.6	252
GCA_000514535.1	*Salinispora pacifica* CNT001	89	5488014	69.8	178.1
GCA_000514575.1	*Salinispora pacifica* CNT003	138	5468952	69.9	248.9
GCA_000514515.1	*Salinispora pacifica* CNT029	45	5233191	69.8	280.2
GCA_000374765.1	*Salinispora pacifica* CNT045	90	5768959	69.6	271.2
GCA_000514495.1	*Salinispora pacifica* CNT084	142	5468756	69.6	272.4
GCA_000374785.1	*Salinispora pacifica* CNT124	55	5160007	69.8	558
GCA_000383575.1	*Salinispora pacifica* DSM 45547	15	5445911	69.8	227.5
GCA_000378825.1	*Salinispora pacifica* DSM 45548	10	5096990	69.9	374.3
GCA_000379065.1	*Salinispora pacifica* DSM 45549	37	5194796	69.4	313.2
GCA_000514815.1	*Salinispora pacifica* CNT569	48	5232086	69.2	350.2
GCA_000514415.1	*Salinispora pacifica* CNT603	81	5394462	69.9	79.1
GCA_000375245.1	*Salinispora pacifica* CNT584	83	5216172	69.7	400.6
GCA_000375265.1	*Salinispora pacifica* CNT609	105	5325580	69.6	213.6
GCA_000424925.1	*Salinispora pacifica* CNY239	92	5366811	69.9	157.7
GCF_010671825.1	*Micromonospora terminaliae* TMS7	40	6699733	73.35	322.6
GCF_003626635.1	*Micromonospora musae* MS1-9	34	7069771	72.2	302.4
GCF_003626545.1	*Micromonospora musae* NGC1-4	28	7058875	72.2	231.5
GCF_000297395.2	*Micromonospora lupini* str. Lupac 08	50	7325824	71.9	244.5
GCF_003583405.1	*Micromonospora radicis* AZ1-13	83	5963223	71.9	335.6
GCF_003626655.1	*Micromonospora costi* CS1-12	20	7182425	73	222.9
GCF_016598485.1	*Micromonospora veneta* CAP181T	137	6799777	72.4	330.7

GCF_016741875.1	*Streptomyces coffeae* CA1R205	253	9896405	70.7	939.4
GCF_009755605.1	*Streptomyces typhae* p1417	69	9812178	72	223
GCF_014649775.1	*Streptomyces longisporoflavus* JCM 396	168	9802249	70.5	525.3
GCF_006334995.2	*Streptomyces mimosae* 3MP-10	45	7198341	73.4	297.1
GCF_003024195.1	*Streptomyces dioscori* A217	57	10239468	70.7	269.1
GCF_002237655.1	*Streptomyces* sp. XY006	74	8251847	71.8	288.8
GCF_008973465.1	*Streptomyces* sp. SS1-1	4	7815656	71.8	90.9
GCF_021390935.1	*Streptomyces* 8s0. CC228A	24	6857711	73.6	546.4
GCF_009025935.1	*Streptomyces* 8s0. CC208B	110	6898902	71.8	524.2
GCF_021390895.1	*Streptomyces* 8s0. CC210A	8	7222961	73.4	582.1
GCA_021390875.1	*Streptomyces* 8s0. CC219A	382	7585741	73.5	450.6
GCA_003352875.1	*Pseudomonas fluorescens strain NEP1*	1	6313875	60.6	144.4
GCA_002287825.1	*Pseudomonas moraviensis strain TYU6*	1	6124581	60.3	178.9
GCA_003293395.1	*Pseudomonas thivervalensis strain PLM3*	1	6591188	61.2	211
GCA_000152545.1	*Pseudomonas aeruginosa 2192*	1	6905121	66.2	78.9
CP021986	*Pseudomonas fragi strain DBC*	1	5015937	54.1	216.8
CP022198	*Pseudomonas oryzihabitans strain MS8*	1	5038716	65.5	247.1
CP020369	*Pseudomonas tolaasii strain 2192T*	1	6856683	60.5	207.5
JACCAH000000000	*Pseudomonas composti strain ZKA28*	1	5672870	62.5	412.6
JACCAJ000000000	*Pseudomonas kunmingensis strain ZKA55*	1	4620945	62.8	130.7
GCA_000022145.1	Anaeromyxobacter dehalogenans 2CP-1	1	5029329	74.7	166
GCA_001027285.1	Archangium gephyra	1	12489432	69.4	259.9
GCA_001189295.1	Chondromyces crocatus	1	11388132	68.7	231.5
GCA_000255295.1	Corallococcus coralloides DSM 2259	1	10080619	69.9	230.6
GCA_000024805.1	Haliangium ochraceum DSM 14365	1	9446314	69.5	331.1
GCA_002305855.1	Melittangium boletus DSM 14713	1	9910441	68.4	392.5
GCA_000331735.1	Myxococcus stipitatus DSM 14675	1	10350586	69.2	195.9
GCA_000012685.1	Myxococcus xanthus DK 1622	1	9139763	68.9	109.7
CP070226	Sandaracinus amylolyticus	2	10963797	71.6	431.2
GCA_000067165.1	Sorangium cellulosum So ce56	1	13033779	71.4	346.6
GCA_000380545.1	*Corynebacterium crenatum MT*	48	3346700	53.9	0
GCA_002775095.1	*Corynebacterium striatum strain 2023*	41	2375104	59.5	0
GCA_019288275.1	*Aspergillus niger strain* CBS 554.65	17	40430000	49.6	210
GCA_000149555.1	*Fusarium verticillioides* 7600	37	41840000	48.7	254
GCA_009663055.1	*Fusarium fujikuroi strain CSV1*	105	43720000	47.5	195
GCA_000993785	*Streptomyces xiamenensis 318*	1	5961402	72	174.1
GCF_003675955.1	*Streptomyces rapamycinicus NRRL 5491*	4	12700000	71	201.9
GCA_000203835.1	*Streptomyces coelicolor A32*	1	9055000	71.5	57.5

Supplementary table 2. Pan-genome statistics for *Lentzea* and *Actinokineospora* genera.

	Organism name	No. Core genes	No. accessory genes	No. of unique genes	No. exclusively absent genes
***Lentzea* genomes**	*L. aerocolonigenes* NBRC 13195	3344	6065	45	1
	L. albidocapillata DSM 44073	3344	4450	46	1
	L. albidocapillata NRRL B-24057	3344	4457	51	1
	L. flaviverrucosa CGMCC 4.578	3344	5269	62	1
	L. flaviverrucosa DSM 44664	3344	5268	66	5
	L. aerocolonigenes NRRL B-3298	3344	6068	85	0
	L. sp. CC55	3344	3822	568	11

L. atacamensis DSM 45479	3344	4718	676	8
L. deserti DSM 45480	3344	4849	683	3
L. albida DSM 44437	3344	4470	694	9
L. albidocapillata subsp. violacea DSM 44796	3344	3874	701	22
L. jiangxiensis CGMCC 4.6609	3344	3821	774	22
L. alba NEAU.D13	3344	4865	791	3
L. nigeriaca DSM 45680	3344	4305	804	17
L. waywayandensis DSM 44232	3344	4815	812	7
L. fradie CGMCC 4.3506	3344	3574	843	81
L. cavernae CGMCC 4.7367	3344	4710	871	6
L. kentuckyensis NRRL B-24416	3344	4686	918	40
L flava JCM 3296	3344	4545	935	3
L. pudingi CGMCC 4.7319	3344	4267	954	6
L. terrae NEAU-LZS 42	3344	5080	1016	17
L. xinjiangensis CGMCC 4.3525	3344	3543	1176	47
L. guizhouensis DHS C013	3344	4336	1668	40
L. sp. FXJ1.1311	3344	2450	2561	521

Actinokineospora genomes

**Organism name**	**No. Core genes**	**No. accessory genes**	**No. of unique genes**	**No. exclusively absent genes**
A. enzaensis DSM 44649	1956	3218	2176	18
A. auranticolor YU 961-1	1956	3501	2111	8
A. fastidiosa JCM 3276	1956	2382	2044	85
A. iranica IBRC-M 10403	1956	2467	1691	41
A. bangkoensis DSM 44EHW	1956	2841	1566	24
A. inagensis DSM 44258	1956	3061	1525	23
A. baliensis DSM 45656	1956	3492	1276	5
A. pegani TRM 65233	1956	2557	1263	52
A. sp. PR83	1956	3855	965	9
A. xionganensis HBU206404	1956	3341	935	12
A. cianjurensis DSM 45657	1956	3995	825	6
A. terrae DSM 44260	1956	4103	591	2
A. spheciospongiae EG49	1956	4279	399	1
A. mzabensis CECT 8578	1956	4276	398	1
A. alba IBRC-M 10655	1956	4456	22	0
A. alba CPCC 201030	1956	4461	18	0

A. alba DSM 45114	1956	4452	16	0

Supplementary table 3. AntiSMASH results for *Lentzea* sp. CC55 (only complete BGCs) and GCF associated.

Region	Type	From	To	Most similar known cluster		Similarity	GCF#
Region 8.1	siderophore	70618	83429				2195
Region 13.2	terpene	255867	276793	isorenieratene	Terpene	0.75	2045
Region 17.1	LAP,thiopeptide	29780	57586				2037
Region 18.1	T3PKS	69039	110076	alkyl-O-dihydrogeranyl-methoxyhydroquinones	Terpene + Polyketide	0.57	2520
Region 19.1	RiPP-like	27080	37898	enduracidin	NRP	0.04	2490
Region 19.2	PKS-like,indole,NRPS,T1PKS	146710	250749	fortimicin	Saccharide	0.06	2005,2173,2398,2522
Region 19.3	lanthipeptide-class-ii	351062	373707				2047
Region 22.1	redox-cofactor	140124	162166	lankacidin C	NRP + Polyketide	0.26	2175
Region 27.1	NRPS-like	35700	79563	indigoidine	Saccharide	0.06	2527
Region 27.2	terpene	96938	119082	geosmin	Terpene	1	2528
Region 33.1	NAPAA	2611	36348				2154
Region 34.1	NRPS	66342	111786	dechlorocuracomycin	NRP	0.12	2531
Region 46.1	NRPS	103568	154430	coelichelin	NRP	0.72	2007
Region 47.1	T2PKS,PKS-like,RRE-containing	198168	270647	LL-D49194α1 (LLD) antitumor	Polyketide	0.32	2202
Region 47.2	lanthipeptide-class-iii	284572	307136	Ery-9 / Ery-6 / Ery-8 / Ery-7 / Ery-5 / Ery-4 / Ery-3	RiPP:Lanthipeptide	0.75	2524
Region 54.1	betalactone	38923	69839	platencin	Terpene	0.06	2008
Region 54.2	terpene	92689	113762	2-methylisoborneol	Terpene	0.5	2040
Region 55.1	NRPS-like,PKS-like,T2PKS	126166	238151	xantholipin	Polyketide	0.12	204,2537
Region 55.2	T1PKS	283753	328261	4-Z-annimycin	Polyketide	0.33	2538
Region 57.1	T1PKS	84	50680	pimaricin	Polyketide	0.29	2539
Region 57.2	NRPS-like	52748	109991	napsamycin A-D / mureidomycin A-B	NRP: + Nucleoside	0.13	2540
Region 57.3	NRPS,CDPS,T1PKS	120986	222233	coelibactin	NRP	0.36	2541
Region 57.4	PKS-like,arylpolyene,T1PKS,NRPS	493491	592270	virginiamycin S1	NRP + Polyketide	0.22	2542
Region 57.5	terpene	678213	700280	geosmin	Terpene	1	1918

Region 57.6	T1PKS,NRPS,hglE-KS,amglyccycl	701985	770886	maduropeptin	Polyketide:Iterative typeI	0.03	2544

Supplementary table 4. AntiSMASH results for *Actinokineospora* sp. PR83 (only complete BGCs) and GCF associated.

Region	Type	From	To	Most similar Known cluster		Similarity	GCF#
Region 5.1	NRPS,T1PKS,PKS-like	304	98061	chlorothricin / deschlorothricin	Polyketide:Modular type I + Polyketide + Saccharide	0.09	2301
Region 10.1	NRPS,arylpolyene	22016	91909	atratumycin	NRP	0.26	2065
Region 11.1	CDPS	71619	92335	apramycin	Saccharide	0.06	2303
Region 16.1	transAT-PKS,PKS-like,T1PKS	68105	171100	kalimantacin A	NRP + Polyketide:Modular type I	0.24	2304
Region 17.1	NRPS	5612	69419	gobichelin A / gobichelin B	NRP	0.44	2305
Region 24.1	RiPP-like	69161	81038				2209
Region 24.2	other,PKS-like,amglyccycl,T1PKS	114918	183437	cetoniacytone A antitumor	Other:Cyclitol	0.38	2212
Region 24.3	T1PKS,NRPS-like	267645	360948	streptovaricin	Polyketide	0.65	188,2308
Region 35.1	T2PKS	90495	163028	JBIR-76 / JBIR-77	Polyketide	0.56	2311
Region 38.1	NAPAA	2306	36265	dynemicin A	Polyketide	0.03	2175
Region 40.1	T2PKS	83180	155671	saquayamycin A	Polyketide	0.25	277
Region 59.1	RiPP-like	3596	14405				53
Region 79.2	T2PKS,ectoine	99927	140302	kosinostatin	NRP + Polyketide	0.11	2315
Region 80.1	terpene	78429	99409	oxalomycin B	NRP + Polyketide	0.09	2316
Region 80.2	siderophore	116820	128580	macrotetrolide	Polyketide	0.33	2165
Region 87.1	furan	4405	25403	methylenomycin A	Other	0.14	2318
Region 108.1	T1PKS	1	4644				2234
Region 114.1	T1PKS	1	4864				2234

Supplementary figure 1. KEGG distribution for core, accessory and unique genes for. **A**. *Lentzea* and **B.** *Actinokineospora*.

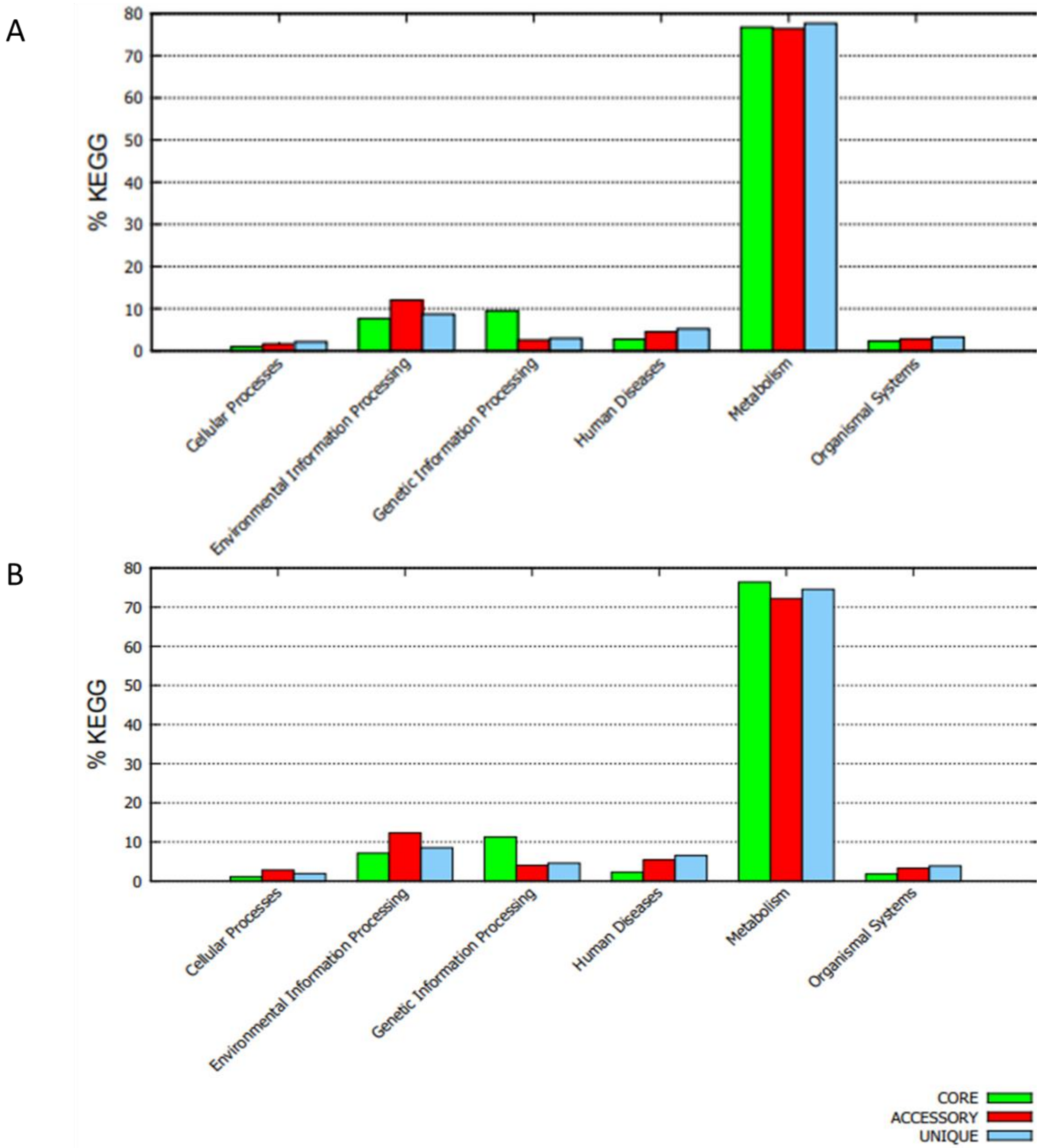

Supplement: Supplementary material 1 [file mgen-9-921-s001.pdf]
